# Gene expression is implicated in the ability of pikas to occupy Himalayan elevational gradient

**DOI:** 10.1371/journal.pone.0207936

**Published:** 2018-12-12

**Authors:** Katherine A. Solari, Uma Ramakrishnan, Elizabeth A. Hadly

**Affiliations:** 1 Department of Biology, Stanford University, Stanford, California, United States of America; 2 Woods Institute for the Environment, Stanford University, Stanford, California, United States of America; 3 National Centre for Biological Sciences, TIFR, Bangalore, India; 4 Program for Conservation Genomics, Stanford University, Stanford, California, United States of America; 5 Center for Innovation in Global Health, Stanford University, Stanford, California, United States of America; INIA, SPAIN

## Abstract

Species are shifting their ranges due to climate change, many moving to cooler and higher locations. However, with elevation increase comes oxygen decline, potentially limiting a species’ ability to track its environment depending on what mechanisms it has available to compensate for hypoxic stress. Pikas (Family Ochotonidae), cold-specialist small mammal species, are already undergoing elevational range shifts. We collected RNA samples from one population of *Ochotona roylei* in the western Himalaya at three sites– 3,600, 4,000, and 5,000 meters–and found no evidence of significant population genetic structure nor positive selection among sites. However, out of over 10,000 expressed transcripts, 26 were significantly upregulated at the 5,000 m site and were significantly enriched for pathways consistent with physiological compensation for limited oxygen. These results suggest that differences in gene expression may play a key role in enabling hypoxia tolerance on this local scale, indicating elevational flexibility that may facilitate successful range shifts in response to climate change.

## Introduction

Current climate trends indicate a continued increase in global mean temperature to as much as 4°C above present temperatures by 2100 [1]. Many species are already shifting their ranges in order to follow their preferred climate [2], with flatland species generally shifting northward, and mountainous species moving to higher elevations [3]. However, moving up in elevation to escape warmer temperatures comes with new environmental stressors, including oxygen deprivation, or hypoxia [4]. Limitations that hypoxia may place on an animal’s immediate ability to move upslope as it responds to warmer conditions have not been explored and likely depend on what hypoxia-compensation mechanisms a population has at its disposal.

Pikas (Order Lagomorpha, Family Ochotonidae) are an ideal model system with which to address this question. Pikas are small mammals related to rabbits and hares. At least 28 extant pika species (*Ochotona* spp.) are known [5]: 2 in North America and the rest in Asia. As many as 15 pika species are found in the Himalayas and/or Tibetan Plateau region [6] which is thought to be the center of origination for the genus [7]. Pikas have a low tolerance for heat [8] and are generally restricted to high latitudes, high elevations, and/or habitats that have cool microclimates. When exposed directly to heat without options for behavioral adaptation, individuals of the American pika (*O*. *princeps*) perish from even brief exposure to temperatures between 25.5–29.4°C [9].

Due to their thermal sensitivity, pikas are expected to be one of the first mammals to respond to climate change, and indeed, the American pika is already showing rapid range shifts in parts of its distribution [10]. As ambient temperatures have warmed, the lower-elevation range margins of American pika populations in the Great Basin have moved up at an average rate of 145 m per decade [10]. Himalayan pika species are at even greater risk of range retraction because the Himalayas are already subjected to rates of temperature change three times the global average [11]. Additionally, the Himalayas are the lowest latitude at which pikas are found.

Many pika species occupy vast elevational ranges. Some species’ elevational ranges span more than 4,000 m and some species live well above 5,000 m [12]. Warming temperatures are likely to continue to make lower-elevation locations within current ranges uninhabitable. Within a species, the ability of lower-elevation pikas to join their higher-elevation neighbors in cooler refugia over a few decades will likely depend on what ‘preadaptive’ mechanisms are already used to tolerate hypoxic stress at different elevations within that species.

Genetic adaptations are known to likely generate variable hypoxia tolerance among pika species. Previous studies assessing genetic adaptations in candidate genes such as hemoglobin [13] and cytochrome c oxidase [14] indicate that lower-elevation pika species do not share many of the unique genotypes that are characteristic of high-elevation species. In fact, not only pikas, but all Himalayan vertebrates studied to date, including indigenous humans [15], yaks (*Bos grunniens*) [16], bar-headed geese (*Anser indicus*) [17], domestic dogs (*Canis lupus familiaris*) [18], snow leopards (*Panthera uncia*) [19], Tibetan grey wolves (*Canis lupus chanco*) [20], and Tibetan wild boars (*Sus scrofa*) [21] display adaptations to tolerate the physiological stress of low-oxygen conditions characteristic of their high-elevation range that are not found in their lower-elevation relatives. However, genetic adaptations occur on an evolutionary time scale that is likely much too slow to keep pace with anthropogenic climate change [22].

Alternatively, alterations in gene expression is a mechanism capable of compensating, at least partially, for the stresses of hypoxia [23,24], and is a mechanism that can occur within hours or days [23,24], easily keeping up with anthropogenic climate change. Plasticity in gene expression in response to chronic hypoxia has been assessed in model organisms such as rats and mice [25,26], and has also been evaluated in a few non-model organisms—deer mice (*Peromyscus maniculatus*) [27–30], rufous-collared sparrows (*Zonotrichia capensis*) [24], Andean human populations [23], and Tibetan pigs (*Sus scrofa*) [31]. In these previous studies of non-model organisms, many significant differences in gene expression between high and low-elevation individuals from distant locations were identified; however, no attempt was made to sample from the same population in order to try to disentangle genetic adaptions, which should be minimal within a population, and gene expression.

**Fig 1:**
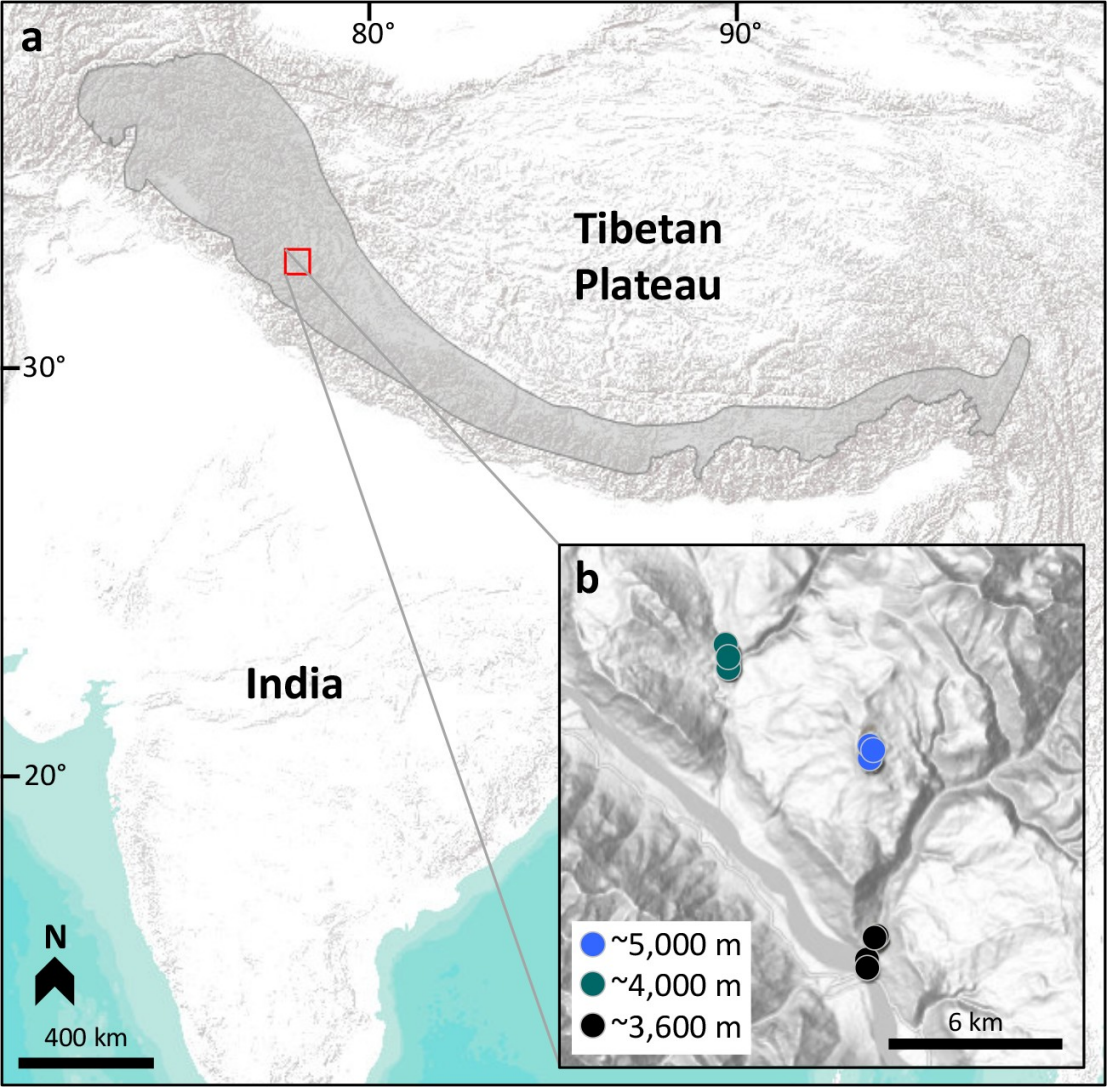
Sampling localities. (a) The complete IUCN distribution of *O*. *roylei* is shown in grey [32]. (b) The three sampling locations are indicated. Reprinted from ArcGIS under a CC BY license, with permission from Esri, original copyright 2009.

In order to get at what is differentiating individuals along an elevational transect at a local level, we targeted the species *Ochotona roylei*, which occurs at elevations from 2,400–5,200 m across the Himalayas (Fig 1) [32]. By spanning such a wide elevational range in sometimes very small geographic areas, this species offered the opportunity to assess differences in hypoxia tolerance at a geographically local scale. Thus we collected samples from pikas across the broadest elevational range possible on one mountain in the Western Himalayas (Fig 1) to quantify genetic structure, genetic adaptation, and gene expression.

In comparing pikas along an elevational transect we expected four possible, non-mutually exclusive, outcomes. Pikas from different elevations could exhibit 1) population structure because they have undergone genetic drift due to a lack of gene flow; 2) differential adaptation at each location apparent through outlier loci under positive selection; 3) differences in gene expression; and 4) no detectable differences at the genetic or transcriptomic levels.

In this study, we address each of these possibilities to determine the relative role of each in generating tolerance to hypoxia in pikas living in this extreme environment. Each mechanism has different implications concerning the response of pikas to climate change. Genetic isolation between elevations (outcome 1) would indicate barriers to dispersal. Genetic adaptations (outcome 2) generally occur much slower than the time frame on which pikas are currently shifting their ranges [22]. If high-elevation individuals within this species have evolved unique genetic adaptations for hypoxia tolerance, then lower-elevation individuals may be genetically unfit to live in high-elevation environments (and vice-versa). Modulation of gene expression (outcome 3) on the other hand, a mechanism that has not yet been explored in pikas, can act on a much shorter time scale [23,24]. Phenotypic plasticity is likely to be one of the most powerful tools that an animal species can have at its disposal to successfully respond to rapid climate change [33]. If high-elevation pikas are dependent on alterations in gene expression to compensate for additional oxygen stress, then this is a mechanism that lower-elevation individuals could potentially be capable of quickly replicating to facilitate range shifts. No detectable differences (outcome 4) would indicate that pikas in this species across this gradient are confronting the challenges of elevation equally, or that the responses to elevation were not detectable at the genomic or transcriptomic level with the samples we collected.

## Materials and methods

### Sample collection

Blood samples were collected from 24 pikas between September 24, 2013 and October 17, 2013 at three different locations along the Southwest side of Mount Kanamo in Spiti Valley, Himachal Pradesh, India (Fig 1). All samples were collected between 32.246° – 32.349° North and 78.000° – 78.056° East (Table 1). Permission to conduct research in this location was granted by the Chief Wildlife Warden of the Himachal Pradesh Forest Department Wildlife Wing under Wildlife Research Study 3853. All samples were obtained by live-trapping using baited Tomahawk traps baited with local red berries and greens as well as fresh fruits and vegetables, such as apples, carrots and corn. Pikas self-transferred from the trap into an anesthetizing chamber through a flexible funnel. Approximately 1 ml of isoflurane was applied to a cotton ball and held in a separate perforated container within a larger anesthetizing chamber. The larger chamber was also perforated in a design that allows adequate air exchange with the outdoor environment. The pika was observed through the clear walls of the chamber and removed for sampling when unresponsive. If the pika became physically active during sampling, it was returned to the chamber for further anesthesia. 500 μL of blood was collected by retro-orbital abrasion using a 100 μL non-heparinized capillary tube. Blood was collected directly into Qiagen RNAprotect animal blood tubes for RNA stabilization. After blood collection, pikas were transferred to an open mesh bag for weighing via a hanging scale and monitored in the bag until they had made a full recovery. Pikas were then released at their point of capture. We experienced no complications during live-trapping and all pikas were successfully sampled and released alive. This study and all procedures described were approved by the Stanford University Administrative Panel on Laboratory Animal Care (APLAC protocol 27547). Using the methods described above, blood collection was performed under isoflurane anesthesia and all efforts were made to minimize discomfort.

**Table 1:** Pika sample localities.

Sample	Collection date	Weight (g)	Elevation (m)	North	East	SRA Accession
BK4	9/25/13	164	3,948	32.34609	78.00051	SAMN06697631
BK1.1	9/25/13	154	4,011	32.34267	78.00072	SAMN06697632
BK1.2	9/25/13	162	4,011	32.34267	78.00072	SAMN06697633
BK1.3	9/25/13	184	4,011	32.34267	78.00072	SAMN06697634
BK8.3	9/26/13	154	3,978	32.34896	77.99937	SAMN06697635
BK6.1	9/24/13	144	3,969	32.34681	78.00021	SAMN06697636
BK6.2	9/24/13	140	3,969	32.34681	78.00021	SAMN06697637
BK6_7	9/26/13	138	4,028	32.3483833	77.9996167	SAMN06697638
BK3_4	9/27/13	-	3,928	32.3445	78.0008167	SAMN06697639
AT1	10/1/13	129	4,931	32.3126667	78.0545	SAMN06697640
AT8.1	10/9/13	124	4,997	32.3178167	78.0536667	SAMN06697641
AT7.1	10/11/13	124	4,978	32.3170667	78.0537833	SAMN06697642
AT7.2	10/11/13	132	4,978	32.3170667	78.0537833	SAMN06697643
AT7.3	10/11/13	124	4,978	32.3170667	78.0537833	SAMN06697644
AT13	10/12/13	141	4,954	32.3168	78.0532167	SAMN06697645
K2	10/15/13	131	3,649	32.2488	78.0520833	SAMN06697646
K1	10/15/13	141	3,643	32.2469833	78.0529333	SAMN06697647
K3.1	10/17/13	110	3,651	32.2572333	78.0553333	SAMN06697648
K3.2	10/17/13	117	3,663	32.2561	78.05525	SAMN06697649
K4.1	10/17/13	149	3,643	32.2563667	78.05595	SAMN06697650

Pikas were visually located at the lowest elevations they occurred and the highest elevations they occurred on the accessible parts of the mountain. Pikas were then sampled from three locations within 12.5 km of each other. Five samples were collected from around 3,600 m near the town of Kaza (~65% oxygen compared to sea-level). Twelve samples were collected from around 4,000 m near Kibber village (~63% oxygen compared to sea-level), approximately 12 km from the 3,600 m site. Seven samples were collected from around 5,000 m near Tinum (~55% oxygen compared to sea-level), approximately 6 km from the 4,000 m site and 7 km from the 3,600 m site (Fig 1). Studies of American pikas show that the microclimates that pikas experience in their underground talus habitat are often significantly different from ambient temperatures and depend on factors such as shade, vegetation, and rock ice features and can also be highly heterogeneous within one talus patch [34,35]. Additionally, pikas are known to use these microclimates to employ precise behavioral thermoregulation to avoid extreme temperatures [36]. Due to this disconnect between the ambient temperature and that experienced by pikas, the role of ambient temperature in gene expression was believed to be negligible compared to that of hypoxia.

Blood was targeted because it is a tissue that could be collected with minimal harm to the animals, and is biologically relevant for responses to limited oxygen. Blood is spread throughout the body and has a relatively quick turnover rate, making it an ideal tissue to gain real-time information of whole body status in response to physiological and environmental pressures [37,38]. Previous studies indicate that 61–80% of protein-coding genes are expressed in the blood transcriptome [37,39].

RNA-stabilized blood samples were stored in a glacier-melt river following collection and transferred to a -80°C freezer within 14 days of collection. Total RNA excluding miRNA (less than 200 nucleotides in length) was extracted and purified from the RNAprotect-stabilized blood samples using the Qiagen RNeasy Protect Animal Blood Kit following the manufacture’s protocol. RNA concentration was assessed using Qubit RNA HS reagents (Thermo Fisher Scientific). RNA integrity was assessed on a bioanalyzer.

### Transcriptome data generation and processing

All samples that had an RNA integrity value greater than 6 and at least 0.5 μg of RNA were used for library preparation. A total of 20 out of the 24 samples passed this quality step and were used to create cDNA libraries to sequence–six samples from the 5,000 m site, nine samples from the 4,000 m site, and five samples from the 3,600 m site (Table 1). These samples were submitted to the Centre for Cellular and Molecular Platforms (C-CAMP) for library preparation and sequencing. Libraries were created using the Illumina TruSeq RNA sample preparation kit. Samples were then sequenced in two 100bp paired-end lanes on an Illumina HiSeq 1000. Twelve samples were sequenced in one lane (AT13, AT7.1, AT7.2, AT8, BK1.3, BK3_4, BK4, BK6.1, K1, K2, K3.1, and K3.2) and eight samples were sequenced in the other (AT1.1, AT7.3, BK1.1, BK1.2, BK6_7, BK6.2, BK8.3, and K4). All raw RNASeq data is available at NCBI Sequence Read Archive (SRA) under accession codes SAMN6697631-SAMN6697650.

Demultiplexed Illumina sequencing reads were corrected for random Illumina sequencing errors using Rcorrector with a *k*-mer size of 31 [40]. *Ochotona spp*. and *Microtus oeconomus* hemoglobin mRNA sequences were downloaded from Genbank (Accession numbers: XM_004589962.1, XM_004589963.1, XM_004596585.1, XM_004596781.1, XM_004589960.2, XM_004589961.2, XM_004596586.2, KC886314.1, KC886313.1, KC886312.1, KC886310.1, JX827174.1, JX827173.1, JQ968413.1, JQ968412.1, DQ839484.1, EF429202.1, JX827171.1, and JX827170.1) and made into a reference file using Bowtie2 [41]. We then aligned the sequence data for each individual to this hemoglobin mRNA reference using Bowtie2 and reads that successfully mapped were removed from the sequence dataset. Reads were treated as single-end to remove adapter sequences and low-quality reads were then removed using Trimmomatic [42]. Reads were then sorted and paired using custom python scripts. Paired reads were then sorted again. Read quality was checked before and after each filtering step using FastQC [43]. The *O*. *princeps* annotated genome and transcriptome were downloaded from NCBI (GCF_000292845.1_OchPri3.0 with 26,240 transcripts). *Ochotona princeps* is the closest relative to *O*. *roylei* with an annotated genome and is approximately 15 million years diverged [44].

### Species verification

The identity of our samples as *O*. *roylei* were confirmed by aligning *MT-CYB* gene sequence from our samples to voucher specimen *MT-CYB* sequence available on Genbank for species in the *Conothoa* subgenus—*O*. *roylei* (JX682573.1), *O*. *macrotis* (JX682574.1), *O*. *rutila* (JX682566.1), *O*. *rufescens* (JF911811.1), *O*. *ladacensis* (JX682569.1), *O*. *forresti* (AF272998.1), *O*. *erythrotis* (AF272999.1), *O*. *koslowi* (AF272993.1)—and *O*. *curzoniae* (JN165307.1) from the *Ochotona* subgenus as an outgroup. Partial *MT-CYB* sequence was recovered from our transcriptomic dataset and sequences were aligned to published sequences using Geneious v7.1.4. We identified the best nucleotide substitution model for our alignment using jModelTest [45] and created a phylogeny using MrBayes [46,47] run for 1 million generations with a sampling frequency of 200 trees and a burn in of 10%. The phylogeny with posterior probabilities was visualized using Geneious v7.1.4

### Variant identification

Variant identification was conducted by following the Broad Institute best practices for variant discovery in RNAseq using GATK [48]. Paired reads for each sample were mapped to the annotated *O*. *princeps* reference genome (GCF_000292845.1_OchPri3.0_genomic.fna) using multi-sample two-pass mapping in STAR aligner [49]. We then used picard tools (http://picard.sourceforge.net) to add read group information, sort, mark duplicates, and index the data for each sample. GATK was used to split reads into exons, trim off any intron sequence, call haplotypes for each sample, and perform joint genotyping of all of the samples together. Base recalibration was not performed because known SNPs are not available for our data; however, variants were filtered using GATK VariantFiltration following GATK recommendations for hard-filtering. GATK VariantFiltration was used to remove variants with a Phred-scaled probably of strand bias (FS) greater than 30, variant quality score normalized by depth of coverage (QD) less than 17, a measure of strand bias (SOR) greater than 3, averaged root mean square mapping quality (MQ) less than 40, read position rank sum (ReadPosRankSum) less than -4, and a depth of coverage (DP) less than 5. We then used VCFtools [50] to remove indels, SNPs missing data for any of the 20 samples, SNPs that were different from the reference but identical across all samples, and singletons. GATK VariantFiltration was then used to remove SNPs in a cluster of three or more within a 30bp window.

### Tests for population structure and selection

SNPs were used to investigate population structure using two Bayesian clustering programs, fastStructure [51] and Admixture v1.3.0 [52] using K = 1 to K = 10. In fastStructure, the optimal K was identified using the “chooseK” script that is part of the fastStructure program. In Admixture, the best K value was determined using cross validation scores. Pair-wise F_st_ values and confidence intervals were calculated using the StAMPP package [53] in R [54] by bootstrapping across loci with 1,000 replicates. We calculated the number of shared and private SNPs in each sampling location using Arlequin 3.5.2.2 [55]. Additionally, we used *MT-CYB* sequences in order to calculate pair-wise F_st_ between sites also using Arlequin 3.5.2.2 [55]. We assessed diversifying selection among the sampling sites using BayeScan v2.1 [56] with a prior odds of 100. PGDSpider v2.1.0.3 [57] was used to create our BayeScan input file.

### Gene expression analysis

The *O*. *princeps* annotated mitochondrial genome was downloaded from Genbank (AJ537415.1) and the 14 annotated mitochondrial genes were extracted and added to the *O*. *princeps* reference transcriptome (GCF_000292845.1_OchPri3.0_rna.fna). This reference transcriptome was indexed and paired reads for each individual were pseudoaligned to this reference using Kallisto v0.42.5 [58]. Kallisto output transcript abundances in transcripts per million (TPM). Sequence based bias correction was implemented and 100 bootstraps were performed on each sample to measure uncertainty in the abundance estimates. Bootstrapping in Kallisto allows us to estimate the probability that a read is assigned to the correct transcript by accounting for technical variance. Transcript abundances and bootstrap values were analyzed in R v.3.2.3 [54] using the package Sleuth v.0.28.0 [59] to identify differentially expressed genes using the Wald test.

Transcripts identified as differentially expressed were run through DAVID v.6.8 [60] where enriched GO categories or KEGG pathways were identified. A heat map summarizing the relative expression and average TPM of each transcript identified as differentially expressed was made using the package gplot v.3.0.1 in R v.3.2.3 [54].

Hemoglobin mRNA sequences were removed to allow for accurate sample quality assessment and data analyses. However, we also performed gene expression analyses on hemoglobin transcripts independently by performing the same procedure described above without the initial removal of hemoglobin transcripts.

## Results

### Species verification

In this study, RNA-stabilized blood samples were successfully collected and sequenced from 20 pikas, representing three collection sites of varying elevations (3,600 m, 4,000 m, and 5,000 m) (Fig 1 and Table 1). We confirmed the species identification of our samples by aligning 664 bp of *MT-CYB* that we recovered sequence for in each of our samples to that of voucher specimens available on Genbank. The favored model for nucleotide substitution in our alignment was TIM2+G based on AIC and TPM2uf+G based on BIC. As in Lecocq et al. [61], models that could not be implemented in MrBayes were replaced by the most similar supported model. In this case, GTR+G was the most similar supported model for both TIM2+G and TPM2uf+G. The phylogeny output from MrBayes with posterior probabilities show that our samples group with *O*. *roylei* (S1 Fig). The *MT-CYB* sequences for all our samples were between 98.3–99.8% identical to the *O*. *roylei* voucher specimen sequence (JX682573.1) and were less than 89.5% identical to any other species in the subgenus to which *O*. *roylei* belongs, the *Conothoa* subgenus.

### Sequencing results

Illumina sequencing resulted in 18.44–55.14 (average 30.81) million reads per sample.

Between 67–89% of the reads for each sample mapped to the hemoglobin mRNA reference and were removed from the sequence dataset. After all filtering steps, between 2.9–14.7 (average 6.3) million paired reads per sample remained.

### Variant identification

Between 65–74% of the final reads for each sample mapped to the reference genome using STAR aligner two-pass mapping [49]. The original variant file contained 2,004,114 sites; however, Genome Analysis Tookit (GATK) filtration steps, indel removal, and the removal of single nucleotide polymorphisms (SNPs) with missing data left 68,788 sites. The reference genome is from a different pika species (*O*. *princeps*), so most of these SNPs were differences between the reference and all of our samples but were not variable within our samples. Once removing these SNPs and singletons, 5,038 SNPs remained and were used in population structure and selection analyses.

### Tests for population structure and selection

Both fastStructure [51] and Admixture v1.3.0 [52] found one population (K = 1) to be the best fit for our SNP dataset. We calculated Weir and Cockerham [62] pairwise F_st_ between each of the three sampling locations based on SNP data and found the F_st_ between the 3,600 m site and the 5,000 m site to be 0.0007 (95%CI: -0.0047–0.0057), between the 4,000 m site and 5,000 m site to be 0.0149 (95%CI: 0.0107–0.0192) and between the 3,600 m site and 4,000 m site to be 0.0184 (95%CI: 0.0135–0.0230). The number of private and shared SNPs between each site showed that the 4,000 m site contained the most unique SNPs and also shared more SNPs with each of the other sites than they did with each other (S2 Fig). We also calculated pairwise F_st_ between each of the three sampling locations based on 664 base pairs of MT-CYB using Arlequin 3.5.2.2 [51] and found no evidence of genetic subdivision between the sampling sites (3,600 m vs. 5,000 m F_st_ = -0.058, p = 0.54; 4,000 m vs. 5,000 m F_st_ = -0.024, p = 0.32; 3,600 m vs. 4,000 m F_st_ = -0.068, p = 0.59). Our BayeScan analysis showed no evidence of diversifying selection among our three sampling sites in any of these SNPs, with no SNPs displaying a q-value less than 0.936 (S3 Fig).

### Gene expression analysis

**Fig 2:**
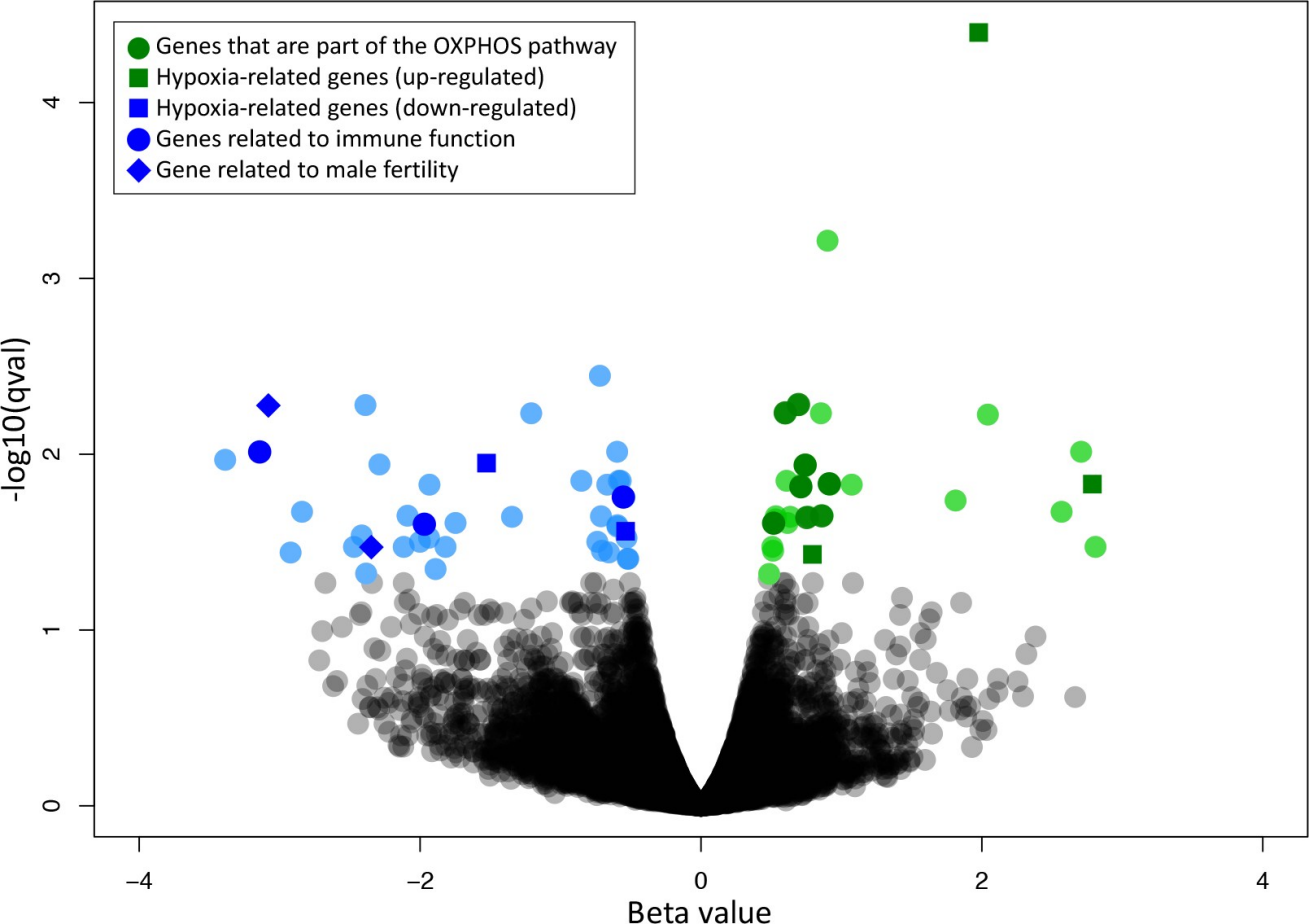
Volcano plot of differential expression between the 5,000 m site and the two lower-elevation sites. Each point is a transcript. The x-axis is the beta value which is the natural log of the fold difference and the y-axis is the–log_10_(qval). The q-value is the multiple test corrected p-value. Colored points are significantly differentially expressed between the high-elevation group and the other locations at a q-value of < 0.05. Transcripts falling above zero on the x-axis (green) are upregulated in the 5,000 m samples and transcripts falling below zero (blue) are down-regulated. Transcripts driving the enrichment of the OXPHOS pathway are shown in dark green circles. Transcripts identified in the text to be of particular interest based on our literature search are also shown with a dark green or dark blue point, with different shapes indicating what each gene is related to.

**Fig 3:**
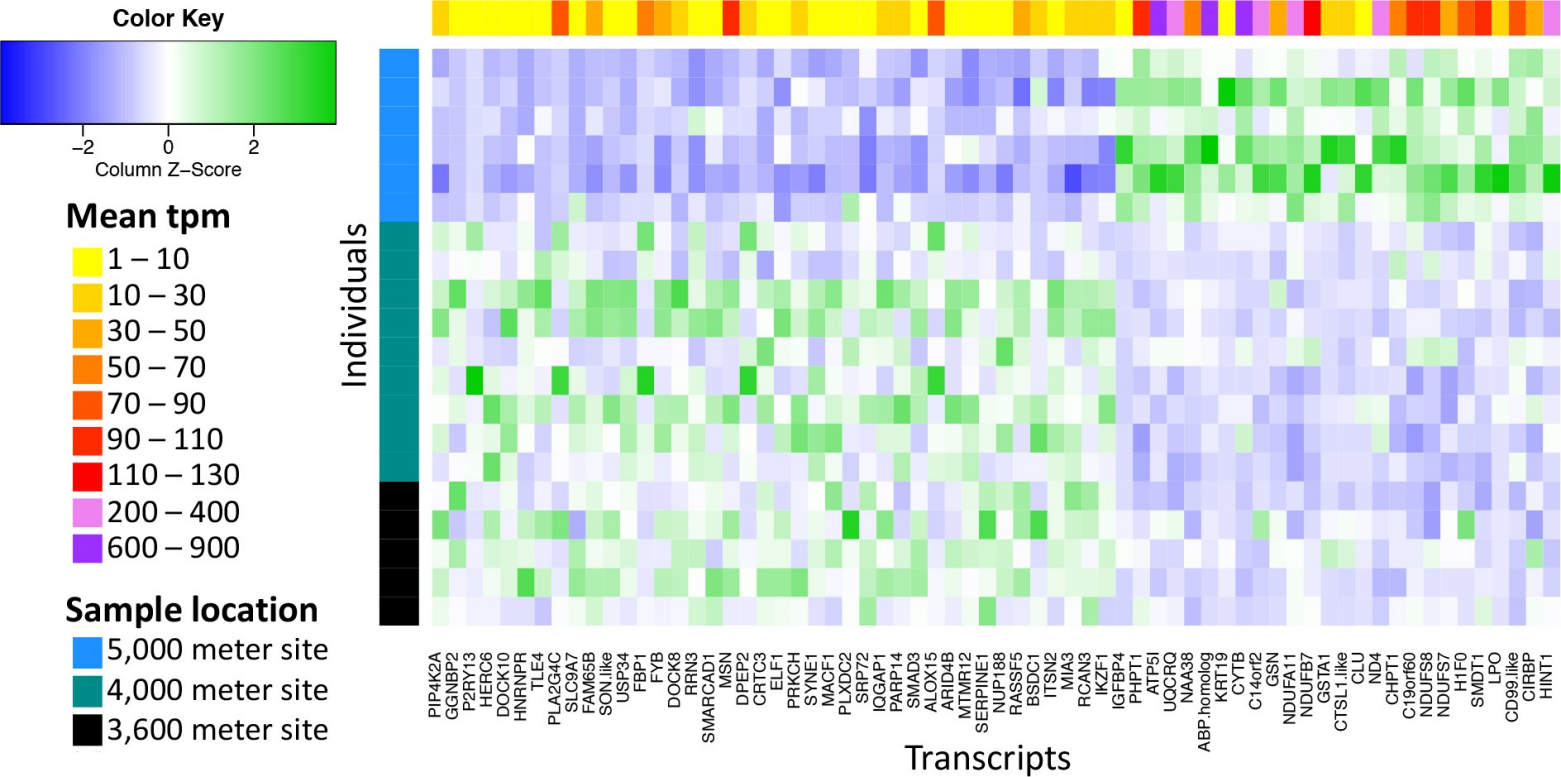
Heat map of transcripts that were significantly differentially expressed in the 5,000 m samples. Each row is an individual, with individuals from the 5,000 m site indicated in blue, the 4,000 m site indicated in teal, and 3,600 m site indicated in black. Each column is a transcript and the mean number of transcripts per million (TPM), a measure of expression level, for each transcript is indicated. In the heat map itself, lower expression is indicated in blue and higher expression is indicated in green.

In each sample, 45–58% (55% average) of all reads mapped to the reference transcriptome with reads mapping to 10,704 of the 26,254 transcripts. After accounting for the false discovery rate, when comparing the sample from the 5,000 m site to samples from the two other locations, 26 transcripts were significantly upregulated in the 5,000 m individuals and 40 transcripts were significantly down-regulated in the 5,000 m individuals (q-value < 0.05) (Fig 2). Our heat map (Fig 3), which also displays transcripts per million (TPM) for each of these transcripts, indicates that transcripts upregulated in the high-elevation group also generally have much higher TPM than the down-regulated transcripts. The set of upregulated and down-regulated genes were each queried in DAVID v.6.8 [60]. The set of upregulated genes (Table 2), 23 of which were successfully annotated with the human genome through DAVID, displayed enrichment for numerous gene ontology (GO) categories and Kyoto Encyclopedia of Genes and Genomes (KEGG) pathways (Table 3). The oxidative phosphorylation KEGG pathway showed the most significant enrichment (Benjamini-Hochberg multiple test correction (BH) p-value = 4.66E-09). DAVID identified six GO categories as being significantly over represented in our list of upregulated genes with BH p-value of < 0.05 (Table 3) all of which are related to the mitochondrial respiratory chain, also known as the mitochondrial electron transport chain. The set of down-regulated genes (Table 4), 39 of which were successfully annotated with the human genome through DAVID, had no enrichment for any GO category or KEGG pathway (BH p-value > 0.27).

**Table 2:** Genes upregulated in the 5,000 m samples.

Transcript ID	Official gene ID^a^	q-value^b^	beta value^c^
XM_004591175.2	IGFBP4	3.98E-5	1.97
XM_004593437.2	PHPT1	6.11E-4	0.90
XM_004599911.2	ATP5I	0.005	0.69
XM_004586401.2	UQCRQ	0.006	0.60
XM_004594778.2	NAA38	0.006	0.85
XM_004595121.1	*ABP homolog*	0.006	2.04
XM_004599151.2	KRT19	0.010	2.71
AJ537415.1_cytb	MT-CYB	0.011	0.74
XM_004584265.2	C14orf2	0.014	0.61
XM_004594967.2	GSN	0.015	1.07
XM_004595601.2	NDUFA11	0.015	0.71
XM_004595838.1	NDUFB7	0.015	0.92
XM_004599448.2	GSTA1	0.015	2.79
XM_004591806.2	*CTSL1-like*	0.018	1.81
XM_004579086.2	CLU	0.021	2.57
AJ537415.1_ND4	MT-ND4	0.022	0.87
XM_012928253.1	CHPT1	0.023	0.53
XM_004595778.1	C19orf60	0.023	0.64
XM_004598226.1	NDUFS8	0.023	0.77
XM_004595916.2	NDUFS7	0.023	0.53
XM_004589519.1	H1F0	0.025	0.62
XM_004589460.2	*SMDT1*	0.034	0.51
XM_004591201.1	LPO	0.034	2.81
XM_012931068.1	*CD99-like*	0.035	0.51
XM_004595641.2	CIRBP	0.037	0.79
XM_012927406.1	HINT1	0.048	0.49

^a^Italicized genes could not be annotated to the human genome in DAVID and are thus not part of the DAVID analysis.

^b^The q-value is the multiple test corrected p-value.

^c^The beta value is the natural log of the fold difference.

**Table 3:** GO terms and KEGG pathways significantly enriched in genes upregulated in the 5,000 m individuals.

Category	Term	Genes	Fold enrichment	Bonferroni p-value	BH p-value^a^	FDR^b^
KEGG_PATHWAY	Oxidative phosphorylation (hsa00190)	NDUFS7, NDUFB7, MT-ND4, NDUFS8, MT-CYB, ATP5I, UQCRQ, NDUFA11	34.64	4.66E-09	4.66E-09	1.91E-07
KEGG_PATHWAY	Parkinson's disease (hsa05012)	NDUFS7, NDUFB7, MT-ND4, NDUFS8, MT-CYB, UQCRQ, NDUFA11	28.39	5.18E-07	2.59E-07	2.12E-05
KEGG_PATHWAY	Non-alcoholic fatty liver disease (NAFLD) (hsa04932)	NDUFS7, NDUFB7, NDUFS8, MT-CYB, UQCRQ, NDUFA11	22.88	3.49E-05	1.16E-05	1.43E-3
KEGG_PATHWAY	Alzheimer's disease (hsa05010)	NDUFS7, NDUFB7, NDUFS8, MT-CYB, UQCRQ, NDUFA11	20.57	5.91E-05	1.48E-05	2.42E-3
GOTERM_CC_DIRECT	mitochondrial respiratory chain complex I (GO:0005747)	NDUFS7, NDUFB7, MT-ND4, NDUFS8, NDUFA11	84.43	1.85E-05	1.85E-05	2.77E-04
KEGG_PATHWAY	Huntington's disease (hsa05016)	NDUFS7, NDUFB7, NDUFS8, MT-CYB, UQCRQ, NDUFA11	18.00	1.14E-04	2.28E-05	4.67E-3
GOTERM_BP_DIRECT	mitochondrial electron transport, NADH to ubiquinone (GO:0006120)	NDUFS7, NDUFB7, MT-ND4, NDUFS8, NDUFA11	81.57	5.07E-05	5.07E-05	3.65E-04
GOTERM_BP_DIRECT	mitochondrial respiratory chain complex I assembly (GO:0032981)	NDUFS7, NDUFB7, MT-ND4, NDUFS8, NDUFA11	63.44	1.41E-04	7.05E-05	1.02E-3
GOTERM_CC_DIRECT	mitochondrial inner membrane (GO:0005743)	NDUFS7, NDUFB7, MT-ND4, MT-CYB, ATP5I, UQCRQ, NDUFA11	13.13	5.37E-04	1.79E-04	8.04E-3
GOTERM_CC_DIRECT	mitochondrion (GO:0005739)	SMDT1, NDUFB7, MT-ND4, NDUFS8, CLU, MT-CYB, ATP5I, C14ORF2, UQCRQ, NDUFA11	6.22	5.27E-04	2.63E-04	7.88E-3
KEGG_PATHWAY	Metabolic pathways (hsa01100)	NDUFS7, NDUFB7, MT-ND4, NDUFS8, MT-CYB, ATP5I, UQCRQ, CHPT1, NDUFA11	4.22	0.00173715	2.90E-04	0.07
GOTERM_MF_DIRECT	NADH dehydrogenase (ubiquinone) activity (GO:0008137)	NDUFS7, NDUFB7, MT-ND4, NDUFS8	67.97	0.001209625	1.21E-3	0.02

^a^Terms are listed in order of BH (Benjamini-Hochberg multiple test correction) p-value.

^b^FDR is the false discovery rate

**Table 4:** Genes down-regulated in the 5,000 m samples.

Transcript ID	Official gene ID^a^	q-value^b^	beta value^c^
XM_004578553.1	PIP4K2A	0.004	-0.72
XM_012928659.1	GGNBP2	0.005	-3.08
XM_012931178.1	P2RY13	0.005	-2.39
XM_012928505.1	HERC6	0.006	-1.21
XM_004576767.2	DOCK10	0.010	-0.60
XM_004592269.2	HNRNPR	0.010	-3.14
XM_004591790.2	TLE4	0.011	-3.39
XM_012930323.1	PLA2G4C	0.011	-1.53
XM_004587867.1	SLC9A7	0.011	-2.29
XM_012929186.1	RIPOR2	0.014	-0.58
XM_012931298.1	*SON-like*	0.014	-0.85
XM_004580288.2	USP34	0.014	-0.57
XM_004591811.2	FBP1	0.015	-1.93
XM_012926508.1	FYB	0.015	-0.67
XM_012928864.1	DOCK8	0.018	-0.54
XM_004587095.2	RRN3	0.021	-2.84
XM_004590564.2	SMARCAD1	0.022	-2.09
XM_004595200.2	MSN	0.023	-0.71
XM_004584170.2	DPEP2	0.023	-1.35
XM_004577918.1	CRTC3	0.025	-1.75
XM_004577448.1	ELF1	0.025	-1.97
XM_004597630.2	PRKCH	0.025	-0.60
XM_004598359.2	SYNE1	0.026	-0.59
XM_004591553.2	MACF1	0.028	-0.53
XM_004578559.2	PLXDC2	0.029	-2.42
XM_004590865.1	SRP72	0.030	-1.94
XM_004577920.2	IQGAP1	0.030	-0.53
XM_004577846.2	PARP14	0.031	-0.74
XM_004577958.1	SMAD3	0.031	-2.00
XM_004594833.1	ALOX15	0.034	-2.12
XM_004578525.2	ARID4B	0.034	-2.35
XM_004583634.2	MTMR12	0.034	-2.47
XM_004587183.2	SERPINE1	0.034	-1.82
XM_004593693.1	NUP188	0.035	-0.71
XM_004578698.1	RASSF5	0.036	-0.66
XM_004591618.2	BSDC1	0.036	-2.92
XM_004582637.2	ITSN2	0.039	-0.52
XM_012930339.1	MIA3	0.039	-0.52
XM_004592246.1	RCAN3	0.045	-1.89
XM_004582218.1	IKZF1	0.048	-2.38

^a^Italicized genes could not be annotated to the human genome in DAVID and are thus not part of the DAVID analysis.

^b^The q-value is the multiple test corrected p-value.

^c^The beta value is the natural log of the fold difference.

We also conducted a literature search on significantly upregulated genes that were not identified in DAVID as playing a role in the over representation of any GO category or KEGG pathway. Interestingly, *GSTA1* plays a role in breaking down toxic products resulting from oxidative stress [63]. As such, the increased expression of this gene is considered to be an adaptation to oxidative stress [63]. The cold-inducible RNA binding protein (*CIRBP*), as evident by its name, displays upregulation in response to mild hypothermia, but is also known to be upregulated in response to hypoxia in human cells [64]. The most significantly upregulated gene, insulin-like growth factor binding protein 4 (*IGFBP4*), regulates growth and development of tissues by negatively regulating insulin-like growth factors (IGFs) and has been seen to be significantly upregulated in response to hypoxia in experiments using human brain cancer cell lines [65].

While there were no enriched GO categories or KEGG pathways in the down-regulated gene set, we also further investigated these genes through a literature search. We found that a number of these genes are known to be down-regulated in response to hypoxia in human cells, such as *PLA2G4C* [66] and *MACF1* [67]. We also found that some of these genes are potentially related to immune response such as *HNRNPR* which positively regulates MHC class 1 expression [68], *DOCK8* which is important in the functioning of natural killer cells and receptor-γt-positive innate lymphoid cells [69,70], and *ELF1* which plays a role in the functioning and development of lymphocytes [71]. Additionally, the second most down-regulated gene, *GGNBP2*, is believed to play a role in spermatogenesis [72], and another significantly down-regulated gene, *ARID4B*, is a positive regulator of male fertility [73].

Analyses were also run comparing low-elevation samples to all other samples, as well as mid-elevation samples to all other samples. The low-elevation samples showed no significantly differentially expressed genes (all q-values > 0.91). The mid-elevation samples displayed one transcript that was marginally significantly different (transcript ID: XM_012929922.1, gene: *ADGRE3*, beta value = 1.18, q = 0.052) with all other transcripts displaying q > 0.33. Additionally, gene expression analysis of the data with hemoglobin transcripts included showed that none of the hemoglobin transcripts were significantly differentially expressed between the 5,000 m site sand the two lower-elevation sites (S1 Table).

## Discussion

Our results indicate that differences in gene expression is likely an important mechanism facilitating hypoxia-tolerance in pikas along the elevational gradient we sampled. After testing for evidence of population structure, differential selection, and differences in gene expression between the sites, we only found evidence of significant differences in gene expression.

We performed multiple analyses to determine if there was any evidence in our data that these three sites could be considered discrete populations. Our SNP analyses indicate that individuals from these three sampling locations are not structured and thus can be considered one population, indicating no substantial barriers to gene flow across this steep elevational gradient. While our results suggest that elevation is not an effective barrier to gene flow, nothing is known about the dispersal distance or effective dispersal barriers for *O*. *roylei*, which has a home range of at least 42 m in diameter [74]. However, in the American pika, dispersal distances of 3 km have been observed for individual pikas [75] and dispersal distances have been estimated with genetic data to be up to 5 km [76] with home ranges of up to about 32 m in diameter [8]. In this study, our three sampling sites are between 6–12 km from each other (Fig 1).

Our SNP analyses also show no signs of differential selection between the sampling locations consistent with the absence of population structure, which implies that gene flow between the sites would be acting against the accumulation of any local adaptations. However, as these transcriptome data are limited to expressed genes, we cannot say for sure that there is not selection occurring in other non-coding regions or in genes that were not captured in our dataset. Further studies assessing selection across the entire genome are necessary to conclusively address differential selection between elevations. Additionally, our limited sample size makes it difficult to estimate the proportion of private vs. shared SNPs and future studies with more samples, and thus more power, could add to our understanding of the mechanisms at play.

The only significant difference between the sampling sites was found in our gene expression analyses. In a field study such as this, we are unable to control for all variables between sites that could impact gene expression; however, confounding environmental variables have been minimized by the proximity of our sampling sites. Additionally, our results show that the functions of the genes undergoing changes in gene expression are consistent with what we would expect in an organism compensating for limited oxygen. We found significant upregulation of genes involved in oxidative phosphorylation and mitochondrial electron transport in the highest elevation individuals. The process of oxidative phosphorylation (OXPHOS) creates 95% of the cell’s energy [77] through the electron transport chain and is an essential cellular process for maintaining the health of the cell and survival of the organism. This process depends on the availability of oxygen, which is used as a terminal electron acceptor; thus limited oxygen, or hypoxia, directly affects cellular viability [78]. Due to the direct effect that hypoxia has on this vital pathway, there have been numerous examples of genes in this pathway undergoing selective pressure in hypoxia-adapted species [14,17,79,80]. Specifically in pikas, Lemay et al. [81] compared the transcriptome of American pikas along an elevational gradient and found different haplotypes of ND5, a mitochondrial gene important to the OXPHOS process, fixed at different elevations.

In fact, similar differences in expression of genes in the OXPHOS pathway have been identified in other species in response to hypoxic stress, however, these studies compared geographically distant high and low-elevation populations. When comparing the gene expression of rufous-collared sparrows living at 2,000 m to those living at 4,100 m in the Peruvian Andes, 187 annotated transcripts were upregulated in the high-elevation individuals and these transcripts were enriched for genes involved in oxidative phosphorylation, oxidative stress response, protein biosynthesis and signal transduction [24]. Cheviron et al. (2008) also found that when high-elevation individuals were brought to low elevation none of these transcripts remained differentially expressed [24], suggesting a within-individual plasticity in gene expression to compensate for elevational stress. Similarly, when comparing gene expression in deer mice from high and low elevations, 221 genes were found to be significantly differentially expressed, with many genes in the OXPHOS pathway upregulated in the high-elevation individuals and linked to elevated oxidative capacity and thermogenic capacity [27,28]. However, in the deer mouse, these changes in gene expression persisted even in a low-elevation common garden but were lost in the F1 generation [27]. The current study does not address whether changes in gene expression can occur within an individual, as in the rufous-collared sparrow [24], or if these expression profiles are hardwired within an individual and can only be reset in their progeny, as seen in the deer mouse [27], perhaps indicating genetic or epigenetic regulation of gene expression [82,83].

Our study adds to the evidence that genes in the OXPHOS pathway are upregulated in response to hypoxia and validates the value of utilizing blood in such a study, a tissue that does not require sacrificing specimens as in similar studies. We hope that the methods outlined here may broaden the options available for gene expression studies without requiring lethal sampling and yet still yield rich, biologically meaningful, data.

Additionally, there was no enrichment for any pathways or GO categories in the set of genes significantly down-regulated in the 5,000 m group; however, we did identify a few genes in which down-regulation may indicate a down-regulation of parts of the immune system and fertility. It is physiologically costly to compensate for increased hypoxia, and further exploration of genes identified here may provide insight as to what trade-offs may be taking place.

Previous studies have shown that gene expression profiles can be very different between geographically distant and genetically distinct populations [24,27,28,84]; however, divergence in gene expression increases with greater genetic distance [85]. Our study capitalized on the uniquely precipitous mountains of the Himalayan massif to draw both high and low-elevation samples from one area and has allowed us to begin to tease apart the role of genetic differences versus expression differences in response to hypoxia.

The results of our study indicate that plasticity in gene expression may be a key mechanism in allowing this pika species to live at 5,000 m versus 4,000 or 3,600 m. Changes in gene expression, unlike genetic adaptations, occur on a time scale that can keep pace with rapid climate change [23,24]. Other studies indicate that different pika species have evolved unique adaptations to hypoxia, perhaps specializing each species for the elevational range that it occupies and potentially limiting range movement outside of that elevational range [13,14]. However, this study suggests that, within a species, plasticity in gene expression may also facilitate range movement at a finer scale. We have not investigated the trade-offs that might be involved in this plasticity, however. Thus, while each species, or even populations, may be ideally suited to its general elevational range through genetic adaptations, within a population, plasticity in gene expression may be responsible for facilitating movement within the species’ elevational envelope. This flexibility in elevation is likely to be an important source of resilience for lower-elevation pika populations impacted by climate change, helping to facilitate successful range shifts to higher, cooler, elevations within their species’ elevational range.

## Supporting information

S1 Fig Bayesian phylogenetic hypothesis based on 664bp of MT-CYB. Posterior probability of each node is indicated. Scale bar represents substitutions per nucleotide site.
(TIF)

S2 Fig Venn diagram of shared versus private SNPs for each sampling location. The number of total SNPs found in each site is given next to the site elevation. The number of SNPs in each section is indicated in bold. The percentage of the total SNPs for each site that a section makes up is indicated. The 5,000 m site is shown in light blue, the 4,000 m site is shown in teal, and the 3,600 m site is shown in grey.
(TIF)

S3 Fig Detection of SNPs under selection using Bayescan. Each point corresponds to a SNP in our dataset. F_st_ is plotted against the q-value, where the q-value is the minimum False Discovery Rate at which the SNP would become significant.
(TIF)

S1 Table Results of differential expression analysis of hemoglobin transcripts between the 5,000 m site and the lower-elevation sites. (DOCX)

## References

[1] IPCC. Summary for Policy Makers. Clim Chang 2014 Impacts, Adapt Vulnerability—Contrib Work Gr II to Fifth Assess Rep. 2014; 1–32. 10.1016/j.renene.2009.11.012

[2] Chen I-C, Hill JK, Ohlemüller R, Roy DB, Thomas CD. Rapid range shifts of species associated with high levels of climate warming. Science. 2011;333: 1024–1026. 10.1126/science.1206432 21852500 10.1126/science.1206432

[3] Guralnick R. Differential effects of past climate warming on mountain and flatland species distributions: a multispecies North American mammal assessment. Glob Ecol Biogeogr. 2007;16: 14–23. 10.1111/j.1466-8238.2006.00260.x

[4] Zhou D, Haddad GG. Genetic analysis of hypoxia tolerance and susceptibility in *Drosophila* and humans. Annu Rev Genomics Hum Genet. 2013;14: 25–43. 10.1146/annurev-genom-091212-153439 23808366 10.1146/annurev-genom-091212-153439 PMC12990993

[5] Lissovsky AA. Taxonomic revision of pikas *Ochotona* (Lagomorpha, Mammalia) at the species level. Mammalia. 2014;78: 199–216. 10.1515/mammalia-2012-0134

[6] IUCN. The IUCN Red List of Threatened Species. Version 2016–3. [Internet]. 2016 [cited 1 Feb 2017]. Available: http://www.iucnredlist.org

[7] Niu Y, Wei F, Li M, Liu X, Feng Z. Phylogeny of pikas (Lagomorpha, *Ochotona*) inferred from mitochondrial cytochrome b sequences. Folia Zool. 2004;53: 141–155.

[8] Smith AT, Weston ML. Ochotona princeps. Mamm Species. 1990;352: 1–8.

[9] Smith AT. The distribution and dispersal of pikas: influences of behavior and climate. Ecology. 1974;55: 1368–1376.

[10] Beever EA, Ray C, Wilkening JL, Brussard PF, Mote PW. Contemporary climate change alters the pace and drivers of extinction. Glob Chang Biol. 2011;17: 2054–2070. 10.1111/j.1365-2486.2010.02389.x

[11] IPCC. IPCC Fourth Assessment Report (AR4) IPCC 2007;1: 976 Available: http://www.ipcc.ch/pdf/assessment-report/ar4/wg2/ar4-wg2-spm.pdf

[12] Smith AT, Xie Y. A Guide to the Mammals of China Princeton, New Jersey: Princeton University Press; 2008.

[13] Tufts DM, Natarajan C, Revsbech IG, Projecto-Garcia J, Hoffmann FG, Weber RE, et al Epistasis constrains mutational pathways of hemoglobin adaptation in high-altitude pikas. Mol Biol Evol. 2015;32: 287–98. 10.1093/molbev/msu311 25415962 10.1093/molbev/msu311 PMC4298171

[14] Solari KA, Hadly EA. Evolution for extreme living: variation in mitochondrial cox genes correlated with elevation in pikas (genus *Ochotona*). Integr Zool. 2018; 10.1111/1749-4877.12332 29851233

[15] Yi X, Liang Y, Huerta-Sanchez E, Jin X, Cuo ZXP, Pool JE, et al Sequencing of 50 human exomes reveals adaptation to high altitude. Science. 2010;329: 75–78. 10.1126/science.1190371 20595611 10.1126/science.1190371 PMC3711608

[16] Qiu Q, Zhang G, Ma T, Qian W, Wang J, Ye Z, et al The yak genome and adaptation to life at high altitude. Nat Genet. 2012;44: 6–11. 10.1038/ng.2343 22751099 10.1038/ng.2343

[17] Scott GR, Schulte PM, Egginton S, Scott ALM, Richards JG, Milsom WK. Molecular evolution of cytochrome c oxidase underlies high-altitude adaptation in the bar-headed goose. Mol Biol Evol. 2011;28: 351–363. 10.1093/molbev/msq205 20685719 10.1093/molbev/msq205

[18] Gou X, Wang Z, Li N, Qiu F, Xu Z, Yan D, et al Whole-genome sequencing of six dog breeds from continuous altitudes reveals adaptation to high-altitude hypoxia. Genome Res. 2014;24: 1308–1315. 10.1101/gr.171876.113 24721644 10.1101/gr.171876.113 PMC4120084

[19] Cho YS, Hu L, Hou H, Lee H, Xu J, Kwon S, et al The tiger genome and comparative analysis with lion and snow leopard genomes. Nat Commun. 2013;4: 2433 10.1038/ncomms3433 24045858 10.1038/ncomms3433 PMC3778509

[20] Zhang W, Fan Z, Han E, Hou R, Zhang L, Galaverni M, et al Hypoxia adaptations in the grey wolf (*Canis lupus chanco*) from Qinghai-Tibet Plateau. PLoS Genet. 2014;10: e1004466 10.1371/journal.pgen.1004466 25078401 10.1371/journal.pgen.1004466 PMC4117439

[21] Li M, Tian S, Jin L, Zhou G, Li Y, Zhang Y, et al Genomic analyses identify distinct patterns of selection in domesticated pigs and Tibetan wild boars. Nat Genet. 2013;45: 1431–1438. 10.1038/ng.2811 24162736 10.1038/ng.2811

[22] Quintero I, Wiens JJ. Rates of projected climate change dramatically exceed past rates of climatic niche evolution among vertebrate species. Ecol Lett. 2013;16: 1095–1103. 10.1111/ele.12144 23800223 10.1111/ele.12144

[23] Appenzeller O, Minko T, Qualls C, Pozharov V, Gamboa J, Gamboa A, et al Chronic hypoxia in Andeans; are there lessons for neurology at sea level? J Neurol Sci. 2006;247: 93–99. 10.1016/j.jns.2006.03.021 16733057 10.1016/j.jns.2006.03.021

[24] Cheviron ZA, Whitehead A, Brumfield RT. Transcriptomic variation and plasticity in rufous-collared sparrows (*Zonotrichia capensis*) along an altitudinal gradient. Mol Ecol. 2008;17: 4556–4569. 10.1111/j.1365-294X.2008.03942.x 18986500 10.1111/j.1365-294X.2008.03942.x

[25] Baze MM, Schlauch K, Hayes JP. Gene expression of the liver in response to chronic hypoxia. Physiol Genomics. 2010;41: 275–288. 10.1152/physiolgenomics.00075.2009 20103700 10.1152/physiolgenomics.00075.2009 PMC2869108

[26] Mosqueira M, Willmann G, Zeiger U, Khurana TS. Expression profiling reveals novel hypoxic biomarkers in peripheral blood of adult mice exposed to chronic hypoxia. PLoS One. 2012;7 10.1371/journal.pone.0037497 22629407 10.1371/journal.pone.0037497 PMC3358260

[27] Cheviron ZA, Connaty AD, McClelland GB, Storz JF. Functional genomics of adaptation to hypoxic cold-stress in high-altitude deer mice: transcriptomic plasticity and thermogenic performance. Evolution (N Y). 2014;68: 48–62. 10.1111/evo.12257 24102503 10.1111/evo.12257 PMC4399701

[28] Cheviron ZA, Bachman GC, Connaty AD, McClelland GB, Storz JF. Regulatory changes contribute to the adaptive enhancement of thermogenic capacity in high-altitude deer mice. Proc Natl Acad Sci. 2012;109: 8635–8640. 10.1073/pnas.1120523109 22586089 10.1073/pnas.1120523109 PMC3365185

[29] Scott GR, Elogio TS, Lui MA, Storz JF, Cheviron ZA. Adaptive modifications of muscle phenotype in high-altitude deer mice are associated with evolved changes in gene regulation. Mol Biol Evol. 2015;32: 1962–1976. 10.1093/molbev/msv076 25851956 10.1093/molbev/msv076 PMC4592356

[30] Velotta JP, Jones J, Wolf CJ, Cheviron ZA. Transcriptomic plasticity in brown adipose tissue contributes to an enhanced capacity for nonshivering thermogenesis in deer mice. Mol Ecol. 2016;25: 2870–2886. 10.1111/mec.13661 27126783 10.1111/mec.13661

[31] Jia C, Kong X, Koltes JE, Gou X, Yang S, Yan D, et al Gene co-expression network analysis unraveling transcriptional regulation of high-altitude adaptation of Tibetan pig. PLoS One. 2016;11 10.1371/journal.pone.0168161 27936142 10.1371/journal.pone.0168161 PMC5148111

[32] Smith AT, Bhattacharyya S. *Ochotona roylei*. In: The IUCN Red List of Threatened Species 2016 [Internet]. 2016 [cited 22 Jan 2017] p. e.T41268A45184591. Available: 10.2305/IUCN.UK.2016-3.RLTS.T41268A45184591.en

[33] Solari KA, Frank HK, Frishkoff LO, Hsu JL, Kemp ME, Mychajliw AM, et al Opportunity for some, extinction for others: the fate of species in the Anthropocene. Evol Ecol Res. 2016;17: 787–813.

[34] Varner J, Dearing MD. The importance of biologically relevant microclimates in habitat suitability assessments. PLoS One. 2014;9: e104648 10.1371/journal.pone.0104648 25115894 10.1371/journal.pone.0104648 PMC4130583

[35] Millar CI, Westfall RD. Distribution and climatic relationships of the American pika (*Ochotona princeps*) in the Sierra Nevada and western Great Basin, U.S.A.; periglacial landforms as refugia in warming climates. Arctic, Antarct Alp Res. 2010;42: 493–496. 10.1657/1938-4246-42.4.493

[36] MacArthur RA, Wang LCH. Behavioral thermoregulation in the pika *Ochotona princeps*: a field study using radiotelemetry. Can J Zool. 1974;52: 353–358. 4819475 10.1139/z74-042

[37] Huang Z, Gallot A, Lao NT, Puechmaille SJ, Foley NM, Jebb D, et al A nonlethal sampling method to obtain, generate and assemble whole blood transcriptomes from small, wild mammals. Mol Ecol Resour. 2016;16: 150–162. 10.1111/1755-0998.12447 26186236 10.1111/1755-0998.12447

[38] Mohr S, Liew CC. The peripheral-blood transcriptome: new insights into disease and risk assessment. Trends Mol Med. 2007;13: 422–432. 10.1016/j.molmed.2007.08.003 17919976 10.1016/j.molmed.2007.08.003

[39] Liew CC, Ma J, Tang HC, Zheng R, Dempsey AA. The peripheral blood transcriptome dynamically reflects system wide biology: a potential diagnostic tool. J Lab Clin Med. 2006;147: 126–132. 10.1016/j.lab.2005.10.005 16503242 10.1016/j.lab.2005.10.005

[40] Song L, Florea L. Rcorrector: efficient and accurate error correction for Illumina RNA-seq reads. Gigascience. GigaScience; 2015;4: 10.1186/s13742-015-0089-y 26500767 10.1186/s13742-015-0089-y PMC4615873

[41] Langmead B, Salzberg SL. Fast gapped-read alignment with Bowtie 2. Nat Methods. 2012;9: 357–359. 10.1038/nmeth.1923 22388286 10.1038/nmeth.1923 PMC3322381

[42] Bolger AM, Lohse M, Usadel B. Trimmomatic: a flexible trimmer for Illumina sequence data. Bioinformatics. 2014;30: 2114–2120. 10.1093/bioinformatics/btu170 24695404 10.1093/bioinformatics/btu170 PMC4103590

[43] Andrews S. FastQC: a quality control tool for high throughput sequence data [Internet]. 2010 Available: www.bioinformatics.babraham.ac.uk/projects/fastqc

[44] Ge D, Zhang Z, Xia L, Zhang Q, Ma Y, Yang Q. Did the expansion of C 4 plants drive extinction and massive range contraction of micromammals? Inferences from food preference and historical biogeography of pikas. Palaeogeogr Palaeoclimatol Palaeoecol. Elsevier B.V.; 2012;326–328: 160–171. 10.1016/j.palaeo.2012.02.016

[45] Darriba D, Taboada GL, Doallo R, Posada D. JModelTest 2: More models, new heuristics and parallel computing. Nature Methods. 2012 p. 772 10.1038/nmeth.2109 22847109 10.1038/nmeth.2109 PMC4594756

[46] Huelsenbeck JP, Ronquist F. MrBayes: Bayesian inference of phylogeny. Bioinformatics. 2001;17: 754–5. 10.1093/bioinformatics/17.8.754 11524383 10.1093/bioinformatics/17.8.754

[47] Ronquist F, Huelsenbeck JP. MrBayes 3: Bayesian phylogenetic inference under mixed models. Bioinformatics. 2003;19: 1572–1574. 10.1093/bioinformatics/btg180 12912839 10.1093/bioinformatics/btg180

[48] Van der Auwera GA, Carneiro MO, Hartl C, Poplin R, del Angel G, Levy-Moonshine A, et al From fastQ data to high-confidence variant calls: the genome analysis toolkit best practices pipeline. Curr Protoc Bioinforma. 2013;43: 1–33. 10.1002/0471250953.bi1110s43 25431634 10.1002/0471250953.bi1110s43 PMC4243306

[49] Dobin A, Davis CA, Schlesinger F, Drenkow J, Zaleski C, Jha S, et al STAR: ultrafast universal RNA-seq aligner. Bioinformatics. 2013;29: 15–21. 10.1093/bioinformatics/bts635 23104886 10.1093/bioinformatics/bts635 PMC3530905

[50] Danecek P, Auton A, Abecasis G, Albers CA, Banks E, DePristo MA, et al The variant call format and VCFtools. Bioinformatics. 2011;27: 2156–2158. 10.1093/bioinformatics/btr330 21653522 10.1093/bioinformatics/btr330 PMC3137218

[51] Raj A, Stephens M, Pritchard JK. FastSTRUCTURE: variational inference of population structure in large SNP data sets. Genetics. 2014;197: 573–589. 10.1534/genetics.114.164350 24700103 10.1534/genetics.114.164350 PMC4063916

[52] Alexander DH, Novembre J, Lange K. Fast model-based estimation of ancestry in unrelated individuals. Genome Res. 2009;19: 1655–1664. 10.1101/gr.094052.109 19648217 10.1101/gr.094052.109 PMC2752134

[53] Pembleton LW, Cogan NOI, Forster JW. StAMPP: an R package for calculation of genetic differentiation and structure of mixed-ploidy level populations. Mol Ecol Resour. 2013;13: 946–952. 10.1111/1755-0998.12129 23738873 10.1111/1755-0998.12129

[54] R-Core-Team. R: a language and environment for statistical computing [Internet]. Vienna, Austria: R Foundation for Statistical Computing; 2014 Available: http://www.r-project.org/

[55] Excoffier L, Lischer HEL. Arlequin suite ver 3.5: a new series of programs to perform population genetics analyses under Linux and Windows. Mol Ecol Resour. 2010;10: 564–567. 10.1111/j.1755-0998.2010.02847.x 21565059 10.1111/j.1755-0998.2010.02847.x

[56] Foll M, Gaggiotti O. A genome-scan method to identify selected loci appropriate for both dominant and codominant markers: a Bayesian perspective. Genetics. 2008;180: 977–993. 10.1534/genetics.108.092221 18780740 10.1534/genetics.108.092221 PMC2567396

[57] Lischer HEL, Excoffier L. PGDSpider: an automated data conversion tool for connecting population genetics and genomics programs. Bioinformatics. 2012;28: 298–299. 10.1093/bioinformatics/btr642 22110245 10.1093/bioinformatics/btr642

[58] Bray NL, Pimentel H, Melsted P, Pachter L. Near-optimal probabilistic RNA-seq quantification. Nat Biotech. 2016;34: 525–527. 10.1038/nbt.3519 27043002 10.1038/nbt.3519

[59] Pimentel HJ, Bray N, Puente S, Melsted P, Pachter L. Differential analysis of RNA-Seq incorporating quantification uncertainty. bioRxiv. 2016; 58164 10.1101/058164 10.1038/nmeth.4324 28581496

[60] Huang DW, Lempicki RA, Sherman BT. Systematic and integrative analysis of large gene lists using DAVID bioinformatics resources. Nat Protoc. 2009;4: 44–57. 10.1038/nprot.2008.211 19131956 10.1038/nprot.2008.211

[61] Lecocq T, Vereecken NJ, Michez D, Dellicour S, Lhomme P, Valterová I, et al Patterns of genetic and reproductive traits differentiation in mainland vs. Corsican populations of bumblebees. PLoS One. 2013;8 10.1371/journal.pone.0065642 23755263 10.1371/journal.pone.0065642 PMC3675023

[62] Weir BS, Cockerham CC. Estimating F-statistics for the analysis of population structure. Evolution (N Y). 1984;38: 1358–1370. 10.2307/2408641 10.1111/j.1558-5646.1984.tb05657.x 28563791

[63] Hayes JD, Mclellan LI. Glutathione and glutathione-dependent enzymes represent a co-ordinately regulated defence against oxidative stress. Free Radic Res. 1999;31: 273–300. 10.1080/10715769900300851 10517533 10.1080/10715769900300851

[64] Wellmann S, Bührer C, Moderegger E, Zelmer A, Kirschner R, Koehne P, et al Oxygen-regulated expression of the RNA-binding proteins RBM3 and CIRP by a HIF-1-independent mechanism. J Cell Sci. 2004;117: 1785–94. 10.1242/jcs.01026 15075239 10.1242/jcs.01026

[65] Minchenko DO, Kharkova AP, Halkin O V., Karbovskyi LL, Minchenko OH. Effect of hypoxia on the expression of genes encoding insulin-like growth factors and some related proteins in U87 glioma cells without IRE1 function. Endocr Regul. 2016;50: 43–54. 10.1515/enr-2016-0008 27560636 10.1515/enr-2016-0008

[66] Flamant L, Toffoli S, Raes M, Michiels C. Hypoxia regulates inflammatory gene expression in endothelial cells. Exp Cell Res. Elsevier Inc.; 2009;315: 733–747. 10.1016/j.yexcr.2008.11.020 19101540 10.1016/j.yexcr.2008.11.020

[67] Udartseva OO, Lobanova M V, Andreeva ER, Buravkov S V, Ogneva I V, Buravkova LB. Acute hypoxic stress affects migration machinery of tissue O2-adapted adipose stromal cells. Stem Cells Int. 2016;2016: 1–16. 10.1155/2016/7260562 PMC5225392 28115943

[68] Reches A, Nachmani D, Berhani O, Duev-Cohen A, Shreibman D, Ophir Y, et al HNRNPR regulates the expression of classical and nonclassical MHC class I proteins. J Immunol. 2016;196: 4967–4976. 10.4049/jimmunol.1501550 27194785 10.4049/jimmunol.1501550

[69] Singh AK, Eken A, Fry M, Bettelli E, Oukka M. DOCK8 regulates protective immunity by controlling the function and survival of RORγt+ ILCs. Nat Commun. 2014;5: 4603 10.1038/ncomms5603 25091235 10.1038/ncomms5603 PMC4135384

[70] Mcmaster ML, Kristinsson SY, Turesson I, Bjorkholm M, Landgren O. DOCK8 Interacts with talin and WASP to regulate natural killer cell vytotoxicity. J Immunol. 2013;190: 3661–3669. 10.4049/jimmunol.1202792 23455509

[71] Ernst P, Hahm K, Trinh L, Davis JN, Roussel MF, Turck CW, et al A potential role for Elf-1 in terminal transferase gene regulation. Mol Cell Biol. 1996;16: 6121–31. Available: http://www.pubmedcentral.nih.gov/articlerender.fcgi?artid=231615&tool=pmcentrez&rendertype=abstract 8887642 10.1128/mcb.16.11.6121 PMC231615

[72] Zhang J, Wang Y, Zhou Y, Cao Z, Huang P, Lu B. Yeast two-hybrid screens imply that GGNBP1, GGNBP2 and OAZ3 are potential interaction partners of testicular germ cell-specific protein GGN1. FEBS Lett. 2005;579: 559–566. 10.1016/j.febslet.2004.10.112 15642376 10.1016/j.febslet.2004.10.112

[73] Wu R-C, Jiang M, Beaudet AL, Wu M-Y. ARID4A and ARID4B regulate male fertility, a functional link to the AR and RB pathways. Proc Natl Acad Sci U S A. 2013;110: 4616–21. 10.1073/pnas.1218318110 23487765 10.1073/pnas.1218318110 PMC3606970

[74] Kawamichi T. Winter behaviour of the Himalayan pika, *Ochotona roylei*. J Fac Sci Hokkaido Univ Ser Ⅴ Ⅰ Zool. 1968;16: 582–594.

[75] Hafner D. Pikas and permafrost: post-Wisconsin historical zoogeography of *Ochotona* in the southern Rocky Mountains, U.S.A. Arct Alp Res. 1994;26: 375–382. 10.2307/1551799

[76] Henry P, Sim Z, Russello MA. Genetic evidence for restricted dispersal along continuous altitudinal gradients in a climate change-sensitive mammal: the American pika. PLoS One. 2012;7: e39077 10.1371/journal.pone.0039077 22720034 10.1371/journal.pone.0039077 PMC3376113

[77] da Fonseca RR, Johnson WE, O’Brien SJ, Ramos MJ, Antunes A. The adaptive evolution of the mammalian mitochondrial genome. BMC Genomics. 2008;9: 10.1186/1471-2164-9-119 18318906 10.1186/1471-2164-9-119 PMC2375446

[78] Lopez-Barneo J, Pardal R, Ortega-Saenz P. Cellular mechanism of oxygen sensing. Annu Rev Physiol. 2001;63: 259–287. 10.1146/annurev.physiol.63.1.259 [pii] 11181957 10.1146/annurev.physiol.63.1.259

[79] Tomasco IH, Lessa EP. Two mitochondrial genes under episodic positive selection in subterranean octodontoid rodents. Gene. 2014;534: 371–378. 10.1016/j.gene.2013.09.097 24113079 10.1016/j.gene.2013.09.097

[80] Xu SQ, Yang YZ, Zhou J, Jing GE, Chen YT, Wang J, et al A mitochondrial genome sequence of the Tibetan antelope (*Pantholops hodgsonii*). Genomics Proteomics Bioinformatics. 2005;3: 5–17. 10.1016/S1672-0229(05)03003-2 16144518 10.1016/S1672-0229(05)03003-2 PMC5172476

[81] Lemay MA, Henry P, Lamb CT, Robson KM, Russello MA. Novel genomic resources for a climate change sensitive mammal: characterization of the American pika transcriptome. BMC Genomics. BMC Genomics; 2013;14: 311 10.1186/1471-2164-14-311 23663654 10.1186/1471-2164-14-311 PMC3662648

[82] Jaenisch R, Bird A. Epigenetic regulation of gene expression: how the genome integrates intrinsic and environmental signals. Nat Genet. 2003;33 Suppl: 245–254. 10.1038/ng1089 12610534 10.1038/ng1089

[83] Pastinen T. Genome-wide allele-specific analysis: insights into regulatory variation. Nat Rev Genet. 2010;11: 533–8. 10.1038/nrg2815 20567245 10.1038/nrg2815

[84] Storz JF, Natarajan C, Cheviron ZA, Hoffmann FG, Kelly JK. Altitudinal variation at duplicated beta-globin genes in deer mice: effects of selection, recombination, and gene conversion. Genetics. 2012;190: 203–216. 10.1534/genetics.111.134494 22042573 10.1534/genetics.111.134494 PMC3249357

[85] Whitehead A, Crawford DL. Neutral and adaptive variation in gene expression. Proc Natl Acad Sci U S A. 2006;103: 5425–30. 10.1073/pnas.0507648103 16567645 10.1073/pnas.0507648103 PMC1414633

