# Supplementary figures and images for: Gene expression is implicated in the ability of pikas to occupy Himalayan elevational gradient
Source: PLoS One. 2018 Dec 12;13(12):e0207936. doi: 10.1371/journal.pone.0207936 (PMC6291101; doi:10.1371/journal.pone.0207936)

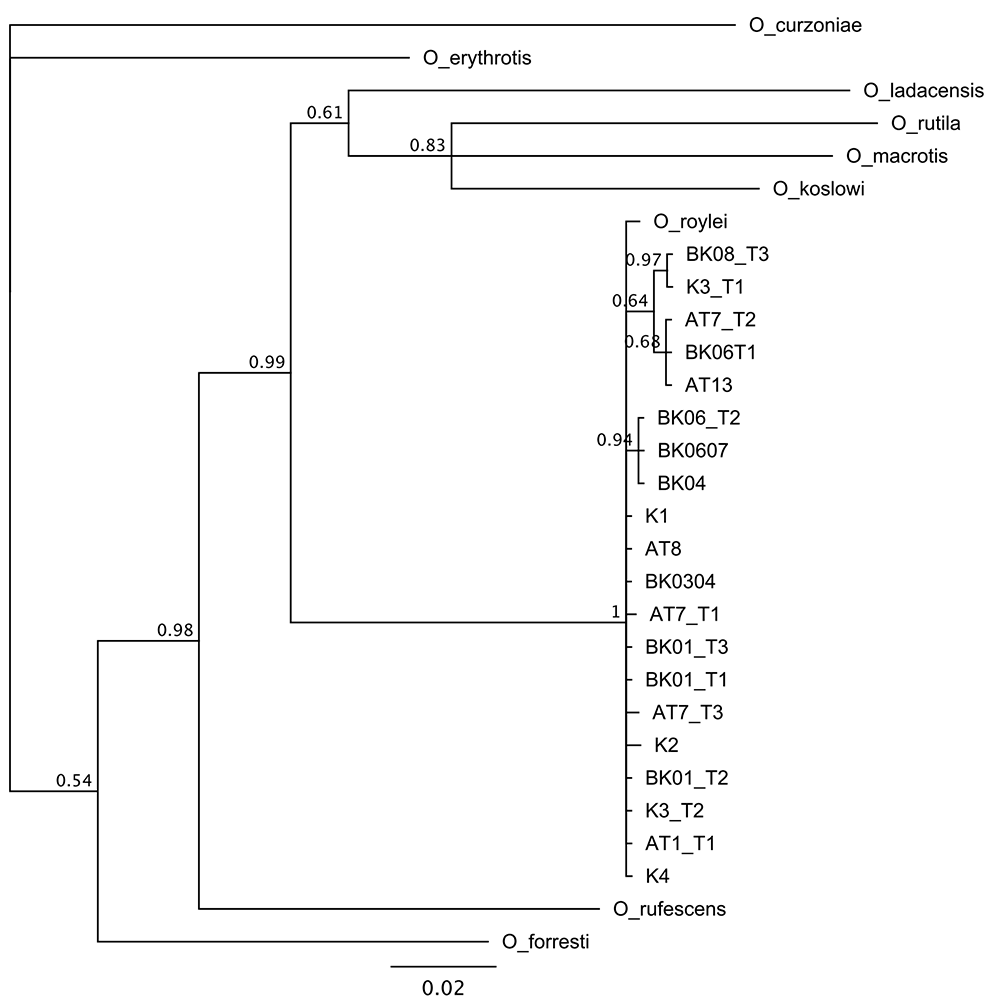

Supplement: S1 Fig — Posterior probability of each node is indicated. Scale bar represents substitutions per nucleotide site. (TIF) [file pone.0207936.s001.tif]

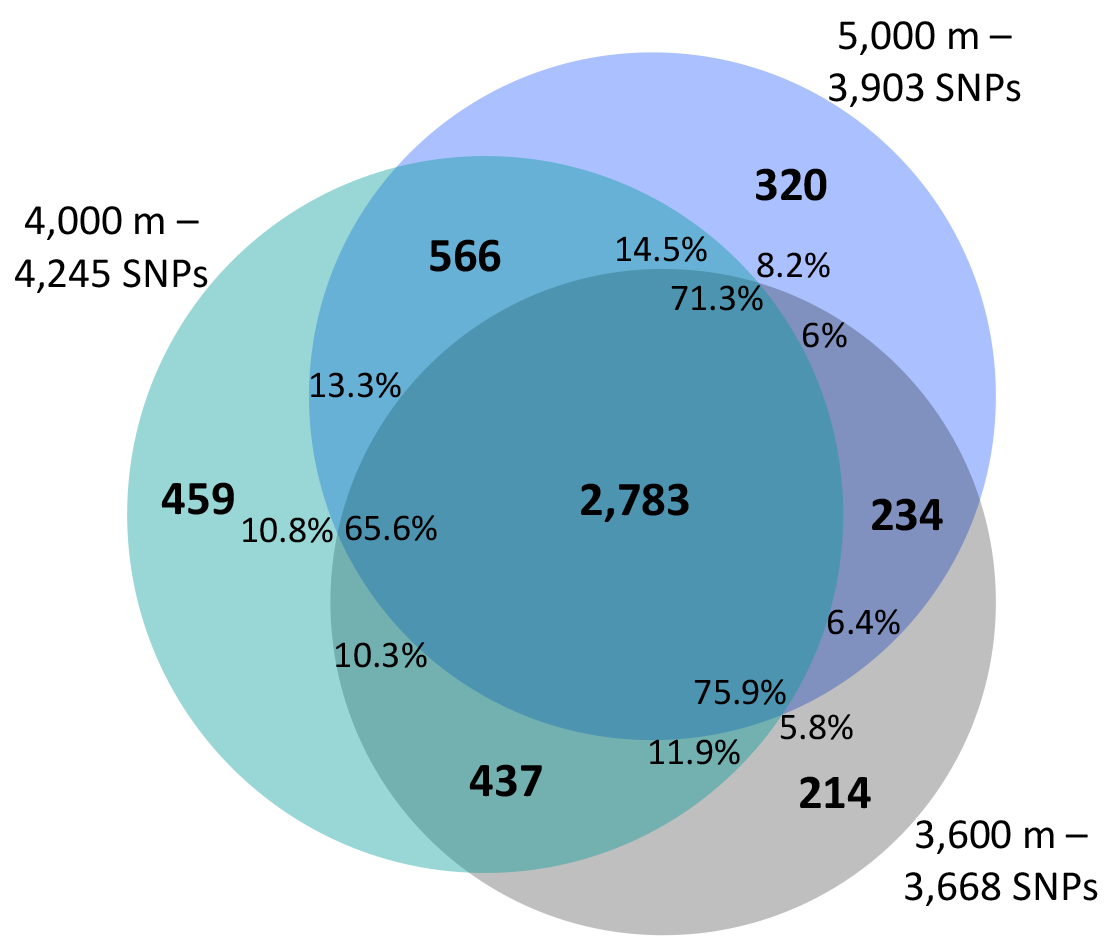

Supplement: S2 Fig — The number of total SNPs found in each site is given next to the site elevation. The number of SNPs in each section is indicated in bold. The percentage of the total SNPs for each site that a section makes up is indicated. The 5,000 m site is shown in light blue, the 4,000 m site is shown in teal, and the 3,600 m site is shown in grey. (TIF) [file pone.0207936.s002.tif]

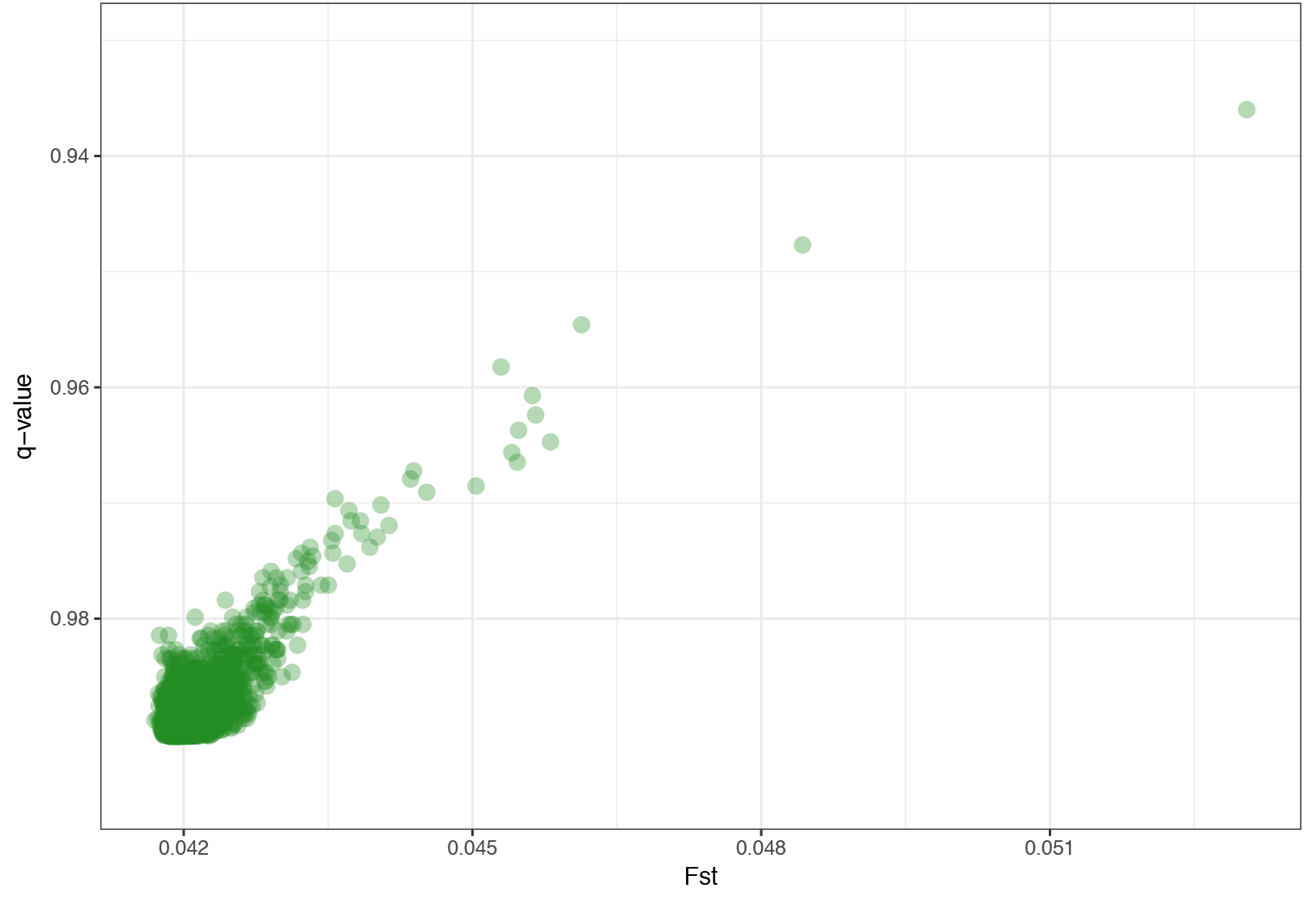

Supplement: S3 Fig — Each point corresponds to a SNP in our dataset. Fst is plotted against the q-value, where the q-value is the minimum False Discovery Rate at which the SNP would become significant. (TIF) [file pone.0207936.s003.tif]
