# Supplementary material for: Gene expression is implicated in the ability of pikas to occupy Himalayan elevational gradient
Source: PLoS One. 2018 Dec 12;13(12):e0207936. doi: 10.1371/journal.pone.0207936 (PMC6291101; doi:10.1371/journal.pone.0207936)
Supplement: S1 Table — (DOCX) [file pone.0207936.s004.docx]

**S1 Table. Results of differential expression analysis of hemoglobin transcripts between the 5,000 m samples and the lower-elevation samples.**

| **Transcript ID** | **Gene annotation^a^** | **q-value**^b^ | **beta value**^c^ |
| --- | --- | --- | --- |
| XM_004596586.2 | hemoglobin, alpha 1 (HBA1) | 0.56 | 0.72 |
| XM_004589961.2 | hemoglobin subunit beta-like (LOC101529624) | 0.70 | 0.42 |
| XM_004589960.2 | hemoglobin subunit beta (LOC101529379) | 0.47 | 0.68 |
| XM_004596781.1 | hemoglobin, theta 1 (HBQ1) | 0.18 | 1.21 |
| XM_004589963.1 | hemoglobin subunit epsilon (LOC101530128) | 0.56 | 0.70 |
| XM_004589962.1 | hemoglobin subunit gamma (LOC101529878) | No transcripts | |
| XM_004596585.1 | hemoglobin subunit zeta (LOC101531846) | No transcripts | |

^a^Gene annotation from the *O. princeps* reference transcriptome. ^b^The q-value is the multiple test corrected p-value. ^c^The beta value is the natural log of the fold difference.
